# Investigating the Blood Oxygenation Level-Dependent Functional MRI Response to a Verbal Fluency Task in Early Stroke before and after Hemodynamic Scaling

**DOI:** 10.3389/fneur.2017.00283

**Published:** 2017-06-19

**Authors:** Veena A. Nair, Ryan V. Raut, Vivek Prabhakaran

**Affiliations:** ^1^Department of Radiology, University of Wisconsin–Madison, Madison, WI, United States; ^2^Neuroscience Training Program, University of Wisconsin–Madison, Madison, WI, United States; ^3^Medical Scientist Training Program, University of Wisconsin–Madison, Madison, WI, United States

**Keywords:** stroke, verbal fluency, functional MRI, resting-state amplitude, language, resting, hemodynamic calibration

## Abstract

**Background and objective:**

Blood oxygenation level-dependent (BOLD) functional MRI (fMRI) has been extensively used as a marker of brain dysfunction and subsequent recovery following stroke. However, growing evidence suggests that straightforward interpretation of BOLD fMRI changes with aging and disease is challenging. In this study, we investigated the effect of calibrating task fMRI data by applying a hemodynamic calibration method using the resting-state fluctuation amplitude (RSFA). Task fMRI responses were obtained during a covert verbal fluency task in a group of early stage stroke patients and matched healthy normal controls.

**Methods:**

Fifteen acute left hemisphere stroke patients (less than 7 days from stroke; aged 44–84 years, average ~64 years) and 21 healthy controls (aged 55–77 years, average ~61 years) were prospectively studied. All subjects completed a 3-min covert verbal fluency task, and a 10-min eyes-closed resting-state fMRI scan, from which the calibration factor (RSFA) was computed. A behavioral measure on the verbal fluency task was also collected outside the scanner. Whole brain activation volumes and region-of-interest (ROI)-wise percent signal change and activation volumes before and after calibration were computed.

**Results:**

Between-group differences in whole brain activation volumes, although statistically significant before calibration failed to be significant after calibration. There were significant within-group differences before and after calibration with RSFA. Statistically significant between-group differences on ROI-wise measures before calibration also significantly reduced after calibration. Exploratory brain-behavior correlations revealed a similar pattern: significant correlations before calibration failed to survive after calibration.

**Discussion and conclusion:**

BOLD fMRI changes with aging and disease is confounded by changes in neurofunctional coupling leading to challenges in the straightforward interpretation of task fMRI results. Application of the hemodynamic calibration using the RSFA technique in the current study appeared to mitigate any differences between stroke and age-matched healthy controls. Our study indicates that estimating neural activity after applying hemodynamic scaling is important for studies of aging and for studies tracking post-stroke changes. We recommend that further investigation of hemodynamic calibration with RSFA in healthy subjects and in stroke in larger samples is necessary.

## Introduction

The blood oxygenation level-dependent (BOLD) contrast in functional MRI (fMRI) studies has been extensively used to study group differences and to relate brain response to overt behavior. The BOLD signal is a function of changes in cerebral blood flow (CBF), volume, and oxygenation. A key challenge, however, in investigating BOLD fMRI task-associated responses in aging and disease is that there may be considerable variability in the responses across subjects depending on the intrinsic vascular reactivity to neural activity (1–3). The mechanisms underlying BOLD blood flow changes are influenced by vascular and neural factors that are poorly understood (4). The strong dependence between CBF and the electrical activity of the brain is accomplished by a process known as neurovascular coupling, whereby increases in neuronal activity is tightly related to corresponding increases in regional CBF (5–8). There is growing evidence that neurovascular coupling is affected in healthy aging and in neurological disorders such as stroke, complicating straightforward interpretation of the BOLD signal.

Several studies have used a breath-hold task in the MR scanner to measure the brain’s cerebrovascular response to a stimulus and a hypercapnic hemodynamic calibration method to eliminate inter-subject variability in task fMRI studies [e.g., Ref. (9–11)]. However, using a breath-hold task with stroke patients poses additional demands on the subjects, potentially leading to compliance issues, and results could be further confounded by inter-subject differences in the extent of performing the breath-hold task. One alternative proposed for the calibration of task fMRI is the resting-state fluctuation amplitude [RSFA; (12)]. Several studies have reported that the amplitude of fluctuations in resting-state fMRI in large part reflects the naturally occurring variations in cardiac rhythm and in respiratory rate and depth and approximates the BOLD response to a hypercapnic challenge (12–15). Calibration using RSFA provides a practically convenient method for removing intra- and inter-subject variability arising from vascular factors. Kannurpatti and colleagues have previously used this method in young and older healthy normals (16). Kannurpatti et al. reported that both intra- and inter-subject variability differences were reduced after calibration with RSFA. Additionally, the amplitude and spatial extent of group activation were lower in the older than in the younger group prior to and after calibration. In the current study, we investigated the efficacy of this technique in stroke. We applied the RSFA calibration to a high-level cognitive fMRI task in early stage stroke patients and in age-matched older healthy control subjects. In order to focus on a relatively homogenous stroke sample, we tested this approach in a sample of early stage left hemisphere stroke patients (mean time since stroke onset less than 7 days). The cognitive task used in this study was a verbal (or phonemic or letter) fluency task in which subjects were required to silently generate words starting with specific letters. This task is known to elicit reliable activation in both older controls and stroke patients and has been extensively used with patient populations (17–19). Along with investigating within- and between-group differences in task-associated BOLD signal amplitude and activation volumes, we also investigated the efficacy of this approach in select regions-of-interest (ROIs) typically activated during performance of this task.

## Materials and Methods

### Participants

The current study is part of an ongoing research study investigating brain reorganization changes following stroke. Data from 15 early left hemisphere stroke patients (mean age = 64 years, range 44–84 years, 11 males, 12 right handed) and 21 healthy controls (mean age = 62.7 years, range 55–77 years, 11 males, 20 right handed) were analyzed for this study. *Inclusion criteria* for the stroke group were patients 18 years or older with ischemic stroke and without a history of major psychiatric illness, confounding neurological disorders, or alcohol or substance abuse. Healthy control group included subjects aged 18 years or older without a history of major psychiatric illness, confounding neurological disorders, vascular risk factors, or alcohol or substance abuse.

The demographic characteristics of all participants are shown in Table 1. Table 2 lists clinical characteristics of the stroke patients in the study. All subjects provided written informed consent and were paid compensation of $75. This study was conducted in accordance with a human subjects’ protocol approved by the University’s Health Sciences Institutional Review Board.

**Table 1:** Demographic characteristics of healthy controls in the study.

Healthy control	Age	M/F	Handedness	Education (years)	Acute stroke	Age	M/F	Handedness	Education (years)
1	71	F	R	18	1	67	M	L	21
2	67	M	R	21	2	58	M	R	16
3	74	F	R	16	3	79	M	R	21
4	61	F	R	12	4	58	F	R	12
5	55	M	R	21	5	70	M	R	20
6	58	M	L	18	6	63	M	L	12
7	60	F	R	16	7	55	M	R	12
8	60	M	R	17	8	62	M	R	16
9	55	F	R	16	9	75	M	R	18
10	59	F	R	21	10	84	M	L	16
11	63	F	R	18	11	44	F	R	16
12	62	F	R	18	12	59	F	R	18
13	56	M	R	22	13	62	F	R	16
14	61	M	R	14	14	62	M	R	14
15	62	F	R	18	15	61	M	R	14
16	77	M	R	14					
17	62	M	R	16					
18	62	F	R	18					
19	63	M	R	16					
20	61	M	R	16					
21	68	M	R	18					

*M, male; F, female; R, right; L, left*.

**Table 2:** Clinical characteristics of early stroke patients.

Patient	Age	Time since stroke (days)	NIH stroke score	Lesion location
1	67	5	0	Left lateral medulla
2	58	5	0	Left corticospinal tract
3	79	6	2	Left MCA
4	58	7	1	Left frontal
5	70	2	2	Left parietal
6	63	2	2	Left putamen
7	55	3	0	Left MCA
8	62	9	0	Left parietal
9	75	6	1	Left occipital
10	84	5	1	Left MCA
11	44	5	7	Left insula, L frontal
12	59	2	2	Left posterior insula, left angular gyrus
13	62	5	2	Left MCA
14	62	12	0	Left paramedian midbrain
15	61	5	0	Left peri thalamic region and left corona radiate

*MCA, middle cerebral artery*.

### Data Acquisition

#### Behavioral Measures

Verbal fluency outside the scanner was assessed by forms of the Controlled Oral Word Association Test (20), which requires subjects to produce words beginning with a letter of the alphabet, “F,” “A,” and “S” in three respective 1-min trials. There is evidence that individuals with vascular risk factors for stroke and stroke patients show impaired performance on the verbal fluency task when compared to age- and education-matched healthy normals (21–23). Each subject was asked to generate as many words as possible to each of the letters “F,” “A,” and “S” per minute. Instructions were given orally and responses recorded on paper by the examiner. Subjects were asked to avoid repetitions, proper nouns, and add-ons (e.g., “please,” “pleasing,” “pleasingly,” etc.). Responses to each letter were recorded, and verbal fluency raw scores were based on the total number of correct responses produced by the participants across the three letter conditions. Normed verbal fluency scores were calculated by subtracting the age and education appropriate control mean score from the individual raw score and dividing by the corresponding SD.

##### MRI Data Acquisition

A 3-T GE whole-body MRI scanner (GE Healthcare, Waukesha, WI, USA) equipped with a 8-channel head coil was used to acquire the MRI images. An axial localizer scan was obtained to verify subject positioning and plan slice acquisition. T1-weighted axial anatomical images were acquired at the beginning of each session using FSPGR BRAVO sequence (TR = 8.132 ms, TE = 3.18 ms, TI = 450 ms, 256 × 256 matrix, 156 slices, flip angle = 12°, FOV = 25.6 cm, slice thickness = 1 mm). Ten minutes, eyes closed, resting-state scans were obtained using single-shot echo-planar T2*-weighted imaging with the following acquisition parameters: TR = 2.6 s, 231 time-points, TE = 22 ms, FOV = 22.4 cm, flip angle = 60°, voxel dimensions 3.5 mm × 3.5 mm, 3.5 mm slice thickness, 40 slices. Functional data were acquired *via* echo-planar T2*-weighted imaging either with the same parameters as the resting or with parameters: TR = 2.0 s, 90 time-points, TE = 22 ms, FOV = 22.4 cm, flip angle = 60°, voxel dimensions 3.75 mm × 3.75 mm, 4.0 mm slice thickness, 40 slices. Each task fMRI scan followed a block design consisting of four 20-s blocks alternating with five 20-s blocks of rest, for a total scan length of 3 min. During the task blocks, subjects viewed the letters “F,” “A,” “S,” and “T” on the screen through the overhead mirror, and during the rest block subjects viewed the letter “REST,” and were instructed to rest and not think of anything in particular. Subjects wore earplugs to attenuate scanner noise, and foam padding was placed around the subject’s head to minimize movement.

### Data Preprocessing

#### fMRI Data

All preprocessing of task and resting fMRI data was performed using the AFNI package (24).

1. *Uncalibrated percent signal change in task* (standard preprocessing of the task fMRI data): for the verbal fluency task, data were first aligned to the anatomical and normalized to standard Montreal Neurological Institute (MNI) space. The first four volumes were discarded to allow for steady-state imaging. Images were then resampled to 3.0 mm isotropic, despiked, volume registered, and spatially smoothed using a 4 mm full-width at half-maximum Gaussian kernel. The time series within each voxel was scaled to percent signal and standard activation maps were computed using a general linear model (GLM) with a canonical gamma variate hemodynamic response function convolved with a boxcar reference waveform and six rigid-body motion parameters and their derivatives regressed. Motion censoring (per TR motion > 0.3 mm) was included in the GLM. The magnitude of task related activation was derived from the beta-weights, which represent the BOLD percent signal change during the task (uncalibrated BOLD amplitude or standard percent signal change). Standard activation maps were also derived using AFNI’s 3dClustSim [*p* < 0.05 (33 voxels)].
2. *Calibrated percent signal change in task*: same preprocessing steps as in (1) were adopted, except that the data were not scaled to percent signal change prior to the GLM. Temporal SD was computed and then divided by the hemodynamic scaling factor (RSFA) which was computed as described below in (4) to get the calibrated BOLD percent signal change (16).
3. *Uncalibrated task activation volume*: same preprocessing steps as in (1). Activation volume was defined as the number of voxels whose percent signal change exceeds the 99th percentile of the uncalibrated null distribution. The computation of this null distribution is described below. To obtain a model free statistical estimate using a data driven approach as implemented by Kannurpatti and colleagues (16), we computed the 99th percentile level of the null distribution, i.e., the distribution obtained from the resting-state scan on a subject-wise basis. To obtain this null distribution, the resting-state data were preprocessed using standard preprocessing steps and scaled to percent signal change and the null distribution computed using a GLM, in which six motion parameters and their derivatives, motion censoring (per TR motion > 0.3 mm) and a stimulus regressor corresponding to the task design extended to match the length of the resting scan, were included (16). To be consistent with the preprocessing of the task data, no additional regressors such as cerebrospinal fluid or white matter signals were included and no band pass filtering was applied. The uncalibrated task-related activation volume was defined as the number of voxels that exceed the 99th percentile of this whole brain uncalibrated null distribution.
4. *Calibrated task activation volume*: to obtain the null distribution after calibration, the temporal SD during task was divided by the RSFA. Following Kannurpatti and Biswal (12) and Kannurpatti et al. (16), RSFA is defined as the temporal SD obtained from resting-state data (i.e., RSFA = ΔBOLD_rest_ = SD_rest_). To compute the threshold for significance, SD of the first half of the resting-state time series (RSFA1) was divided by the SD of the second half (RSFA2) to obtain the ratio in every voxel (this procedure avoided the acquisition of another resting-state scan). The *calibrated task-related activation* volume was defined as the number of voxels that exceed the 99th percentile of this whole brain calibrated null distribution (RSFA1 ÷ RSFA2). The 99th percentile value was also applied to derive activation maps for comparison with pre-calibration maps using the same cluster correction approach.

All preprocessing was done in subject space and then images normalized to MNI space for computing uncalibrated and calibrated BOLD signal amplitude and activation volumes.

#### Selection of ROIs

In order to evaluate the RSFA calibration method at the ROI level, an almost identical process as outlined above was used for computing the BOLD percent signal change and activation volumes with the key difference that the 99th percentile threshold value was computed for each ROI to compute BOLD signal amplitude and activation volumes at an uncorrected significance threshold of *p* < 0.01. Based on a recent meta-analysis of the verbal fluency task, the four selected ROIs included: two regions in the left inferior frontal gyrus (MNI coordinates, −48, 28, 14 and −52, 12, 0), and two regions in the right inferior frontal gyrus (50, 20, 2 and 52, 18, 6) (19).

### Statistical Tests

Chi-squared tests were run to examine group differences on demographic variables of gender and handedness. Independent *t*-tests were applied to compute between-group differences in age, education, and on the raw and normed verbal fluency scores obtained outside the scanner. Bartlett’s test was used to test for homogeneity of variances across the groups. Differences between group means on the imaging variables of whole brain BOLD amplitude and task activation volumes were tested using the unpaired Student’s *t*-test and changes within each group, from pre- to post-calibration, were tested using paired *t*-test (a *p* < 0.05 was considered statistically significant). For ROI-wise comparisons, a value of *p* < 0.01 (=0.05/4 ROIs; Bonferroni correction) was considered statistically significant.

## Results

Chi-squared tests showed no significant differences between the groups on gender (*p* = 0.16) or handedness (*p* = 0.16). There were also no significant differences in age (*p* = 0.65) or education (*p* = 0.20) between stroke patients and healthy controls.

### Behavioral

#### Letter Fluency Task

**Figure 1:**
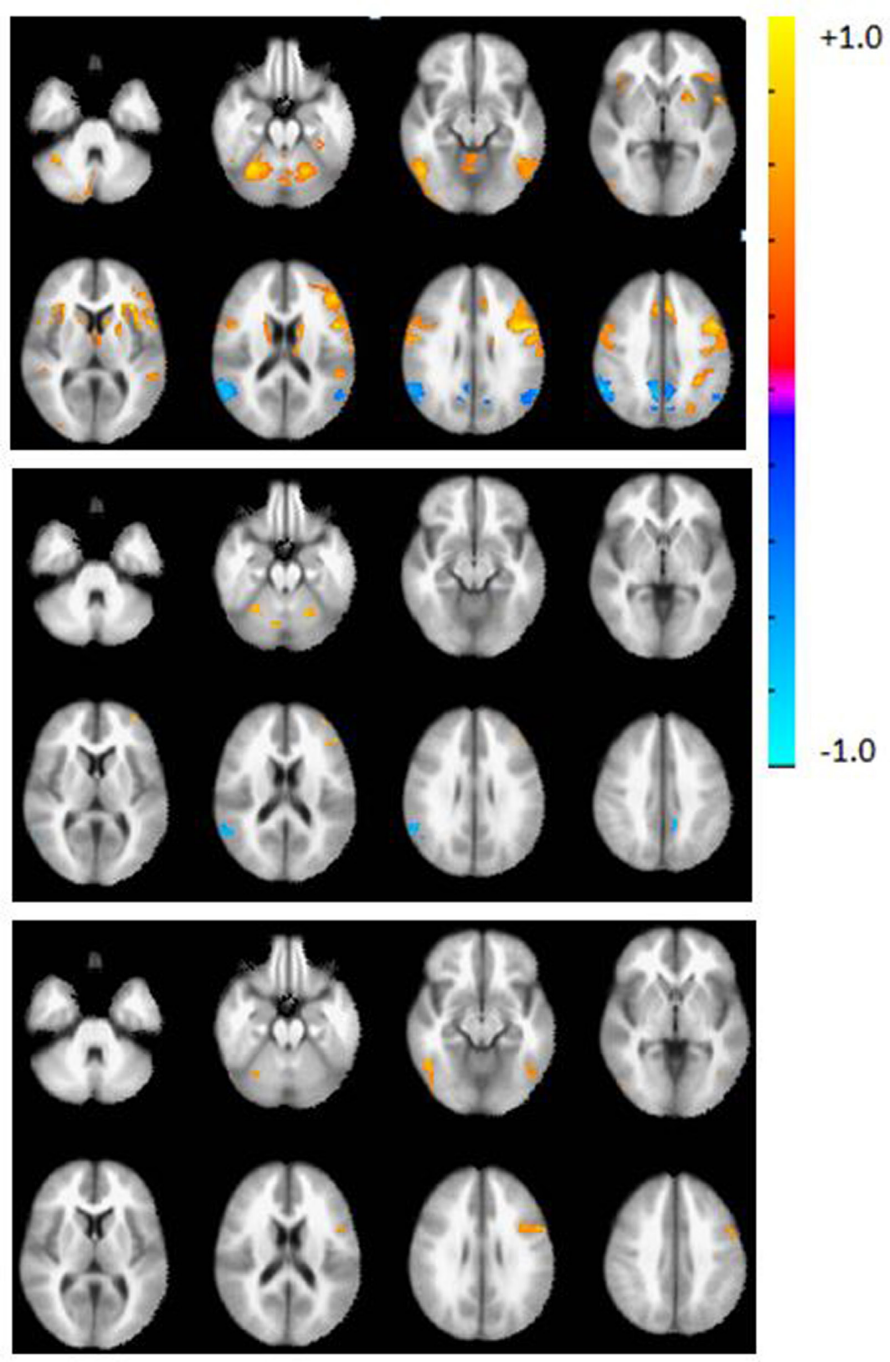
Axial view of the group activation maps before calibration during the verbal fluency task in controls (top), patients (middle), and difference map (controls > patients, bottom panel). Maps are in radiological convention, left = right, *p* < 0.05, corrected using AFNI’s 3dClustSim. Warm colors indicate areas of activation with respect to the rest block; cool colors indicate deactivation with respect to the rest block.

There were significant differences between stroke patients and healthy controls on the letter fluency task raw scores (mean = 27 in acute strokes vs. 51 in healthy controls, *p* < 0.00000) and normed scores (*p* < 0.00000) (Figure 1).

### Imaging Data

Bartlett’s test showed no differences in variance in whole brain activation volume between groups (*p* > 0.11).

#### Pre-Calibration Activation Maps, BOLD Amplitude, and Activation Volumes

**Figure 2:**
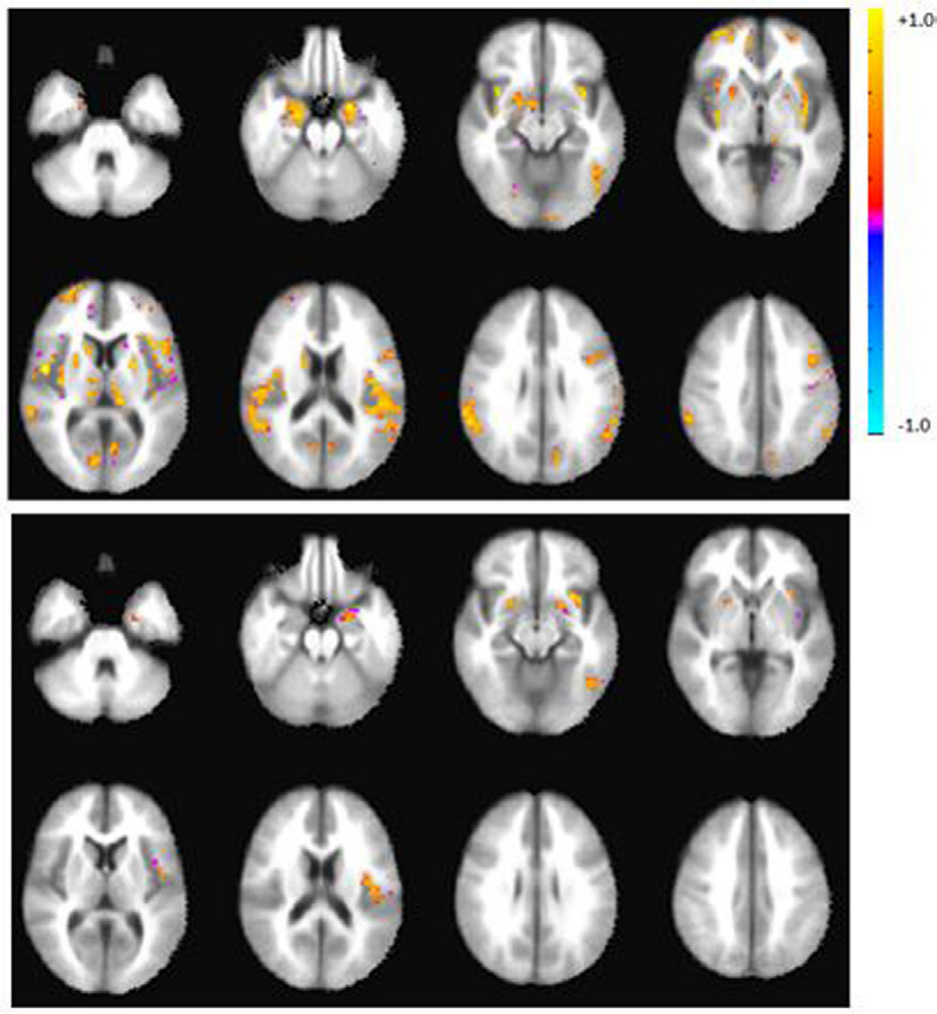
Axial view of the group activation maps after calibration with RSFA during the verbal fluency task in controls (top), patients (bottom panel). Maps are in radiological convention, left = right, *p* < 0.05, corrected. There were no significant group differences after calibration.

Prior to calibration with RSFA, whole brain group activation volumes were significantly smaller in the patient group, compared to the controls during the verbal fluency task (*p* < 0.05). Whole brain activation maps show regions of activation typically found on this task (Figure 2, top). Table 3 lists the top five regions of activation and corresponding MNI coordinates for each group and for the between-group comparison on the verbal fluency task. Controls showed significantly greater activation in the left inferior frontal gyrus, the right inferior temporal gyrus, right cerebellum, and the left inferior occipital gyrus.

**Table 3:** Regions of activation (uncalibrated) in controls and patients on the verbal fluency task.

	Montreal Neurological Institute coordinates					
Controls	*X*	*Y*	*Z*	Region	Voxels	Max intensity *T*
1	+6	−18	+48	L supplementary motor area (SMA)	1,779	7.48
2	−33	60	−30	R cerebellum	994	7.91
3	−30	−21	12	R insula	464	7.28
4	−63	48	18	R superior temporal gyrus	383	−6.34
5	33	42	42	L inferior parietal lobule	356	6.68
**Patients**						
1	−60	+54	+24	R angular gyrus	149	−6.33
2	−27	+57	−21	R cerebellum	114	7.91
3	+45	+9	+54	L postcentral gyrus	87	5.84
4	+45	−33	+24	L inferior frontal gyrus (p. triangularis)	50	6.36
5	+3	−3	+66	L SMA	43	6.95
**Controls > patients**						
1	39	−9	+24	L inferior frontal gyrus (p. opercularis)	144	7.45
2	−51	+54	−12	R inferior temporal gyrus	96	6.44
3	−24	+72	−54	R cerebellum	82	7.69
4	+39	+66	−3	L inferior occipital gyrus	63	4.51
5	−24	+69	−27	R cerebellum	46	7.91

#### Post-Calibration Activation Maps, BOLD Amplitude, and Activation Volumes

**Figure 3:**
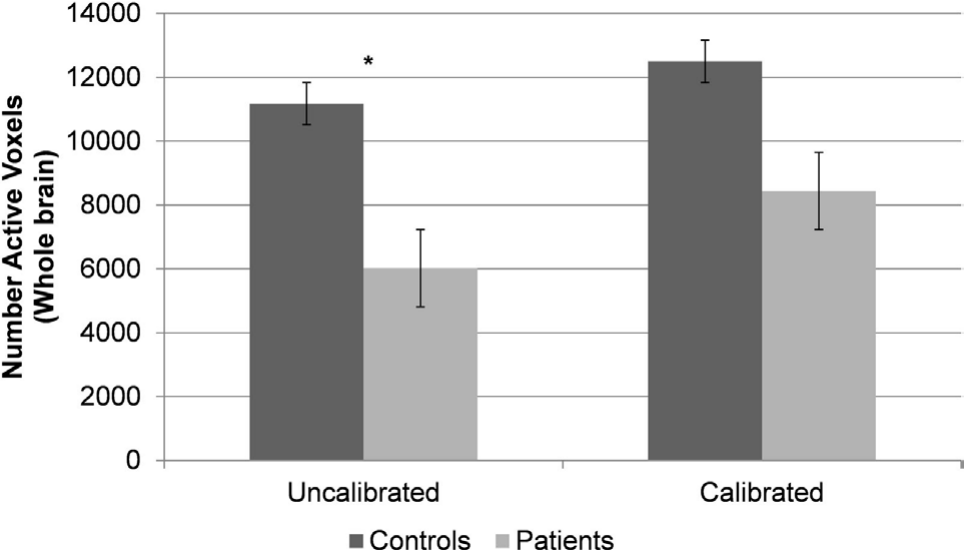
Within- and between-group differences in whole brain activation volumes.

After calibration with RSFA, whole brain group activation volumes remained smaller in the patient group, compared to the controls during the verbal fluency task, but this difference was not statistically significant (*p* = 0.22). Table 4 lists the top five regions of activation and corresponding MNI coordinates for each group on the verbal fluency task. Figure 3 shows within- and between-group differences in whole brain activation volume before and after calibration.

**Table 4:** Regions of activation (calibrated) in controls and patients on the verbal fluency task.

After calibration	Montreal Neurological Institute coordinates					
Controls	*X*	*Y*	*Z*	Region	Voxels	Max intensity *T*
1	30	−24	3	L insula	853	5.16
2	−60	45	27	R supramarginal gyrus	513	5.16
3	45	66	−12	L inferior occipital gyrus	147	4.26
4	−39	−54	−3	R middle frontal gyrus	123	4.26
5	−24	9	−24	R parahippocampal gyrus	104	4.26
**Patients**						
1	33	−9	−9	L insula	75	5.83
2	45	0	9	L rolandic operculum	67	4.12
3	42	63	−15	L fusiform gyrus	47	4.12
4	−30	−18	−12	R insula	44	4.61
5	6	45	−63	L cerebellum	43	4.12

### ROI Comparisons

**Figure 4:**
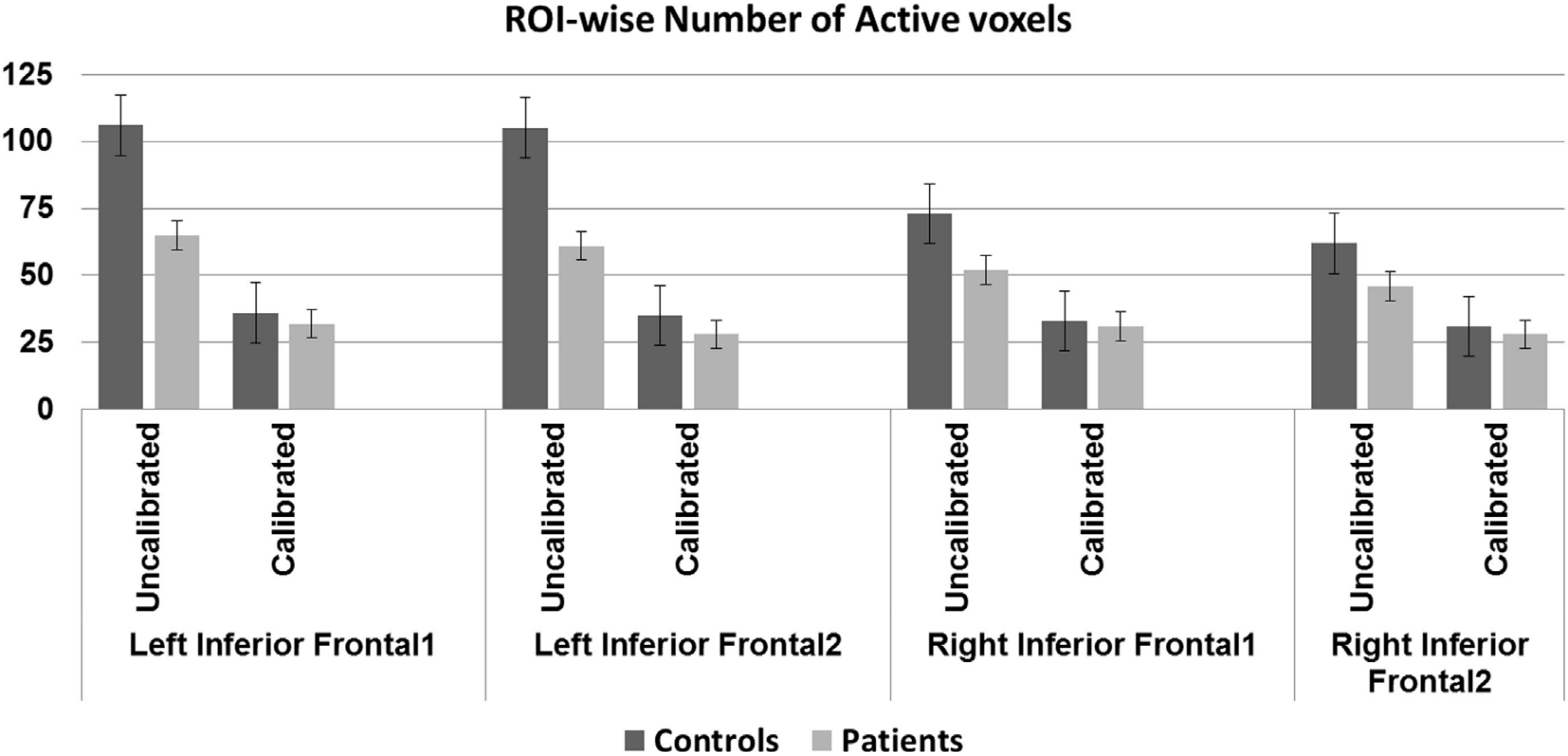
Region-of-interest (ROI)-wise activation volumes—older controls show statistically significant difference before and after calibration in all regions except the right IFG2 (*p* < 0.01, Bonferroni corrected). Patients show statistically significant difference before and after calibration in the left IFG1 (*p* < 0.01, Bonferroni corrected). Significant group difference in the left IFG regions before (*p* < 0.01, Bonferroni corrected) but not after calibration.

Activation volumes reduced in both groups after calibration. Between-group differences were significant prior to calibration in two of four regions but these differences were not significant after calibration. Within-group differences from pre- to post-calibration were significant for three of four regions in controls, and for one region, the left IFG1, in patients (Table 5 and Figure 4).

**Table 5:** Region-of-interest-wise group activation volumes (number of voxels) and *p*-values associated with within- and between-group differences.

	Left inferior frontal 1			Left inferior frontal 2			Right inferior frontal 1			Right inferior frontal 2		
	Uncalibrated	Calibrated	*p*-Value w/in group	Uncalibrated	Calibrated	*p*-Value w/in group	Uncalibrated	Calibrated	*p*-Value w/in group	Uncalibrated	Calibrated	*p*-Value w/in group
Controls	106	36	**8.53606E−05**	105	35	**0.0002**	73	33	**0.008**	62	31	0.05
Patients	65	32	**0.008**	61	28	0.026	52	31	0.120	46	28	0.15
*p*-Value between groups	**0.0056**			**0.0094**			0.204			0.322		

*Significant *p*-values (Bonferroni corrected, *p* < 0.01) are highlighted*.

**Figure 5:**
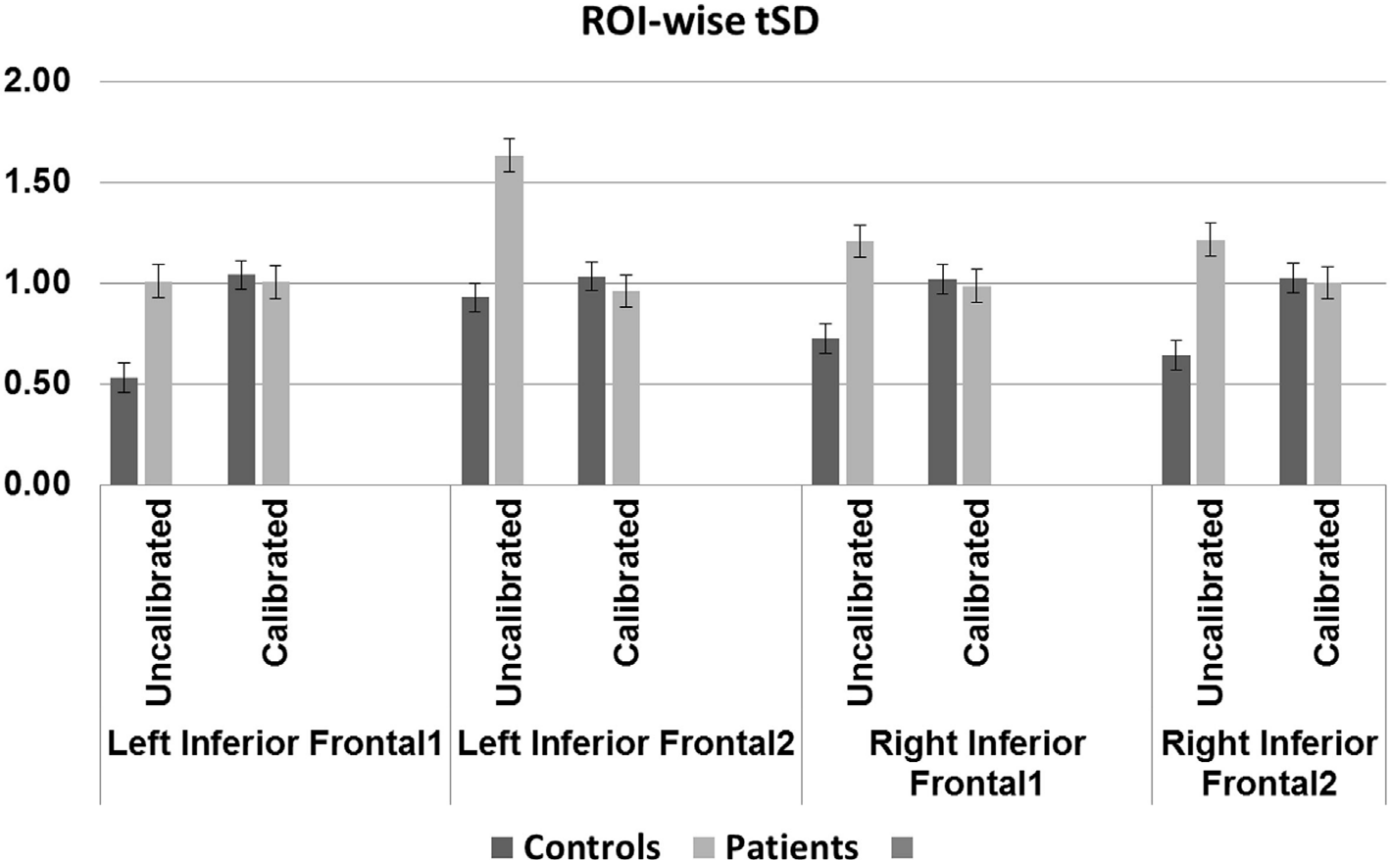
Region-of-interest (ROI)-wise blood oxygenation level-dependent amplitude defined as conventional percent signal change for uncalibrated data and tSD (task) ÷ tSD (rest) (temporal SD of task divided by temporal SD of rest) for calibrated data: older controls show statistically significant difference before and after calibration in all regions except the left IFG2 (*p* < 0.01, Bonferroni corrected). Patients show trend toward significant difference before and after calibration in the left IFG2 (*p* = 0.02). Significant (*p* < 0.01, Bonferroni corrected) group difference in the left IFG regions before but not after calibration.

Blood oxygenation level-dependent amplitude increased post-calibration in controls but not in patients. Between-group differences were, however, significant only prior to calibration in two of four regions; there were no significant differences after calibration in any of the ROIs. Within-group differences from pre- to post-calibration were significant for three of four regions, only in controls (Table 6 and Figure 5).

**Table 6:** Region-of-interest-wise percent signal change and *p*-values (Bonferroni corrected) associated with within- and between-group differences.

	Left inferior frontal 1			Left inferior frontal 2			Right inferior frontal 1			Right inferior frontal 2		
	Uncalibrated	Calibrated	*p*-Value w/in group	Uncalibrated	Calibrated	*p*-Value w/in group	Uncalibrated	Calibrated	*p*-Value w/in group	Uncalibrated	Calibrated	*p*-Value w/in group
Controls	0.53	1.04	**8.53606E−05**	0.93	1.03	0.357	0.72	1.02	**0.007**	0.64	1.03	**0.0008**
Patients	1.01	1.00	0.98	1.63	0.96	0.021	1.21	0.99	0.385	1.22	1.00	0.489
*p*-Value between groups	**0.002**	0.687		**0.006**	0.494		0.034	0.755		0.037	0.820	

*Significant *p*-values (Bonferroni corrected, *p* < 0.01) are highlighted*.

### Brain–Behavior Correlations

Exploratory Pearson correlations between ROI-wise brain measures and verbal fluency normed scores showed trend toward significance only in patients. For activation volume, prior to calibration, Pearson *r* = −0.56, *p* = 0.029 (in the right IFG1), this did not survive significance after calibration. For amplitude, prior to calibration, Pearson *r* = −0.54, *p* = 0.037 (in the right IFG1) and *r* = −0.56, *p* = 0.028 (in the right IFG2) but these did not survive significance after calibration. Note with four ROIs, a *p* < 0.01 would be considered statistically significant here, so *p* < 0.05 here is considered only a trend.

## Discussion

Task fMRI has been extensively used to map between-subject variability in healthy controls and to track post-stroke recovery (23, 25–27). However, several studies have indicated that neurovascular coupling may be compromised with aging in healthy subjects and in patient populations (4, 5, 8, 11). A common assumption routinely made in task fMRI studies is that the amplitude and delay in the hemodynamic response is quite predictable. However, studies have indicated that conventional BOLD analysis techniques may not be sensitive enough for detecting task-associated activity in stroke patients who may be susceptible to changes in neurovascular coupling following both local ischemic changes and diaschisis effects (28). There is a growing effort in the neuroimaging community to understand the effects of stroke on neurovascular coupling, and the possible implications for fMRI studies tracking stroke recovery. Our results, discussed below, however, suggest that application of the RSFA as a hemodynamic calibration factor in patient population needs more investigation before it can be routinely adopted as a hemodynamic scaling factor.

The main objective of this work was to investigate the use of a calibration parameter in the analysis of the effects of stroke on task fMRI BOLD activation. We compared results from stroke patients with a group of age-matched healthy controls before and after calibration. The calibration measure we investigated, “resting-state fluctuation amplitude (RSFA),” derived from a 10-min resting fMRI scan, may be a useful proxy for cerebrovascular reactivity, especially in patient populations, where the patient’s ability to perform a breath-hold task could confound results.

Based on task fMRI studies of language recovery following stroke, it has been proposed that in the early stages there is little activation in the left hemisphere followed by a more bi-hemispheric activation in the sub-acute stage and subsequent restoration of activation in the left hemisphere in the chronic stages of stroke (27). However, what is not known is how much of the reduced activation in the left hemisphere in patients in the early stages is a result of a dysfunctional neurovascular system. Dynamic changes following stroke including structural, vascular, and perfusion changes all contribute to changes in brain activity as measured by BOLD fMRI making it challenging to directly interpret results from this technique in this patient population.

In the current study, there were significant group differences in behavioral performance on the verbal fluency task that is consistent with prior studies (23). Although none of our patients had overt clinical deficits, patients performed poorly on the fluency task. Note, by overt clinical deficits, we mean deficits that would be noted during the clinical team’s evaluation as part of the patient’s standard of care. A review of the patient’s medical records showed that none of the patients included in this study had any documented clinical language deficits. The verbal fluency task is a neuropsychological test that is reported in this study was administered for research purposes and was not done as part of the clinical evaluation. Task fMRI results showed that prior to calibration, controls showed greater whole brain activation compared to stroke patients in the regions of the left inferior frontal gyrus, left occipital gyrus, the right inferior temporal, and cerebellar regions. However, after calibration with RSFA, there were no significant between-group differences. Quantitative comparisons were consistent with this finding with no between-group differences in activation volumes after calibration. For our ROI-wise comparison, we chose standard ROIs typically associated with activation on the verbal fluency task. Notably, both controls and patients showed differences from pre- to post-calibration in activation volumes and BOLD amplitude; however, this effect appears more pronounced in the controls than patients. Within-group changes in activation volume from pre- to post-calibration were greater for the controls (three of four regions) than for stroke patients (one of four regions). This result is consistent with a recent study from our lab (11) in which we used the breath-hold task and observed that the older controls demonstrated significantly impaired CVR compared to younger healthy controls, while stroke patients did not. Within-group changes in amplitude from pre- to post-calibration were greater for the controls (3 of 4 regions) than for stroke patients (0 of 4 regions). Taken together, these findings may suggest that CVR is upregulated in stroke patients in the early stages of stroke.

Similar to the whole brain results, ROI-wise comparisons also showed that significant between-group differences in activation volumes or the BOLD amplitude in the left inferior frontal regions disappeared after calibration. Interestingly, although activation volumes continued to be slightly greater in controls than patients, ROI-wise amplitudes after calibration appeared to be similar in patients and controls. These mixed results therefore indicate that further investigation into these differences with other hemodynamic calibration techniques is warranted (29).

### Methodological Considerations

A basic assumption in fMRI studies investigating group differences is that the two groups have comparable neurovascular coupling. However, in the face of pathology that affect this coupling in one group (e.g., stroke) but not the other (or perhaps a different pathology in the other, e.g., aging) make these comparisons extremely difficult. So when calibration mitigates group differences, as reported in this study, it is difficult to determine if a previously existing difference (in the uncalibrated state) is now obscured by calibration, or calibration merely identified additional regions of activation for both groups, thereby reducing group differences in activation. There may be other factors that may capture the clinical deficit such as functional and structural network connectivity differences between the group, if activation differences do not capture it.

Not only alterations in the cerebral vasculature but also alterations in the complex neurochemical transformation of neural activity to changes in blood flow could also affect the measured BOLD response (30). Healthy “normal” aging is associated with changes in resting CBF and cerebral blood oxygen consumption, even in the absence of gross pathology. In order to address these aspects, we will need to evaluate different calibration methods in a large homogenous sample of subjects as well as take into account secondary factors such as comorbidities, medications, and silent white matter lesions. However, this is beyond the scope of the current study. The current study is simply a demonstration of the application of the RSFA calibration technique in a “fairly” homogenous sample of stroke patients with patients all having left hemisphere strokes. There appears to be a difference in the analysis results when we have applied calibration vs. when no calibration was applied, and therefore there is a contribution of “a resting state fluctuation amplitude calibration method” to the analysis. How useful this method is in identifying true group differences require further studies with carefully controlled and large sample sizes.

Our study has several limitations, including relatively small sample size and studying both cortical and subcortical stroke patients. Additionally, our study differs from previous analyses using RSFA in that we have taken extra steps to reduce motion artifact, which may have confounded previous reports. Nonetheless, we found mixed results when scaling with RSFA. RSFA itself as a calibration factor is relatively novel but is known to be sensitive to neural activity and head motion in addition to physiological changes such as variations in breathing (31); thus, correspondence with other CVR estimates should continue to be studied, particularly when proper motion correction is applied. The strengths of our study are that, we had a fairly homogenous group of stroke patients, with all left hemisphere lesions, and subjects in the two groups were matched on gender, age, and handedness.

## Conclusion

Because resting fMRI can be easily acquired during a MR scan session, we recommend that hemodynamic calibration of BOLD fMRI activation in stroke patients with the RSFA should be further explored and the nature of the RSFA should be further explored as well since it may potentially be a convenient calibration factor. Although studies with larger sample sizes are needed to evaluate the effect of such calibration on brain–behavior relationships, our current study provides a first step in the application of hemodynamic calibration using the RSFA technique.

## Ethics Statement

This study was carried out in accordance with the recommendations of the University of Wisconsin-Madison Health Sciences Institutional Review Board, with written informed consent from all subjects. All subjects gave written informed consent in accordance with the Declaration of Helsinki. The protocol was approved by the UW-Madison IRB.

## Author Contributions

VN was involved in recruitment, data collection, data analysis, and writing. RR was involved in data analysis and writing. VP is the lead PI on the study and supervised all aspects of this work.

## Conflict of Interest Statement

The authors have no conflicts of interest to report, as this research was conducted in the absence of commercial and financial relationships that might compromise the integrity of the results reported herein.

## References

[1] Ances BM Liang CL Leontiev O Perthen JE Fleisher AS Lansing AE Effects of aging on cerebral blood flow, oxygen metabolism, and blood oxygenation level dependent responses to visual stimulation. Hum Brain Mapp (2009) 30:1120–32. 10.1002/hbm.20574 18465743 PMC2810490

[2] Liu P Hebrank AC Rodrigue KM Kennedy KM Section J Park DC Age-related differences in memory-encoding fMRI responses after accounting for decline in vascular reactivity. Neuroimage (2013) 78:415–25. 10.1016/j.neuroimage.2013.04.053 23624491 PMC3694392

[3] Fabiani M Gordon BA Maclin EL Pearson MA Brumback-Peltz CR Low KA Neurovascular coupling in normal aging: a combined optical, ERP and fMRI study. Neuroimage (2014) 85(Pt 1):592–607. 10.1016/j.neuroimage.2013.04.113 23664952 PMC3791333

[4] Tsvetanov KA Henson RN Tyler LK Davis SW Shafto MA Taylor JR The effect of ageing on fMRI: correction for the confounding effects of vascular reactivity evaluated by joint fMRI and MEG in 335 adults. Hum Brain Mapp (2015) 36:2248–69. 10.1002/hbm.22768 25727740 PMC4730557

[5] D’Esposito M Zarahn E Aguirre GK Rypma B. The effect of normal aging on the coupling of neural activity to the bold hemodynamic response. Neuroimage (1999) 10:6–14. 10.1006/nimg.1999.0444 10385577

[6] Iadecola C. The pathobiology of vascular dementia. Neuron (2013) 80:844–66. 10.1016/j.neuron.2013.10.008 24267647 PMC3842016

[7] Grady CL Garrett DD. Understanding variability in the BOLD signal and why it matters for aging. Brain Imaging Behav (2014) 8:274–83. 10.1007/s11682-013-9253-0 24008589 PMC3922711

[8] Veldsman M Cumming T Brodtmann A. Beyond BOLD: optimizing functional imaging in stroke populations. Hum Brain Mapp (2015) 36:1620–36. 10.1002/hbm.22711 25469481 PMC6869358

[9] Thomason ME Burrows BE Gabrieli JD Glover GH. Breath holding reveals differences in fMRI BOLD signal in children and adults. Neuroimage (2005) 25:824–37. 10.1016/j.neuroimage.2004.12.026 15808983

[10] Handwerker DA Gazzaley A Inglis BA D’Esposito M. Reducing vascular variability of fMRI data across aging populations using a breathholding task. Hum Brain Mapp (2007) 28:846–59. 10.1002/hbm.20307 17094119 PMC6871393

[11] Raut RV Nair VA Sattin JA Prabhakaran V. Hypercapnic evaluation of vascular reactivity in healthy aging and acute stroke via functional MRI. Neuroimage Clin (2016) 12:173–9. 10.1016/j.nicl.2016.06.016 27437178 PMC4939388

[12] Kannurpatti SS Biswal BB. Detection and scaling of task-induced fMRI-BOLD response using resting state fluctuations. Neuroimage (2008) 40:1567–74. 10.1016/j.neuroimage.2007.09.040 18343159 PMC10664765

[13] Kalcher K Boubela RN Huf W Biswal BB Baldinger P Sailer U RESCALE: voxel-specific task-fMRI scaling using resting state fluctuation amplitude. Neuroimage (2013) 70:80–8. 10.1016/j.neuroimage.2012.12.019 23266702 PMC3591255

[14] Golestani AM Chang C Kwinta JB Khatamian YB Jean Chen J. Mapping the end-tidal CO2 response function in the resting-state BOLD fMRI signal: spatial specificity, test-retest reliability and effect of fMRI sampling rate. Neuroimage (2015) 104:266–77. 10.1016/j.neuroimage.2014.10.031 25462695

[15] Dubois J Adolphs R. Building a science of individual differences from fMRI. Trends Cogn Sci (2016) 20:425–43. 10.1016/j.tics.2016.03.014 27138646 PMC4886721

[16] Kannurpatti SS Motes MA Rypma B Biswal BB. Increasing measurement accuracy of age-related BOLD signal change: minimizing vascular contributions by resting-state-fluctuation-of-amplitude scaling. Hum Brain Mapp (2011) 32:1125–40. 10.1002/hbm.21097 20665721 PMC3310892

[17] Gaillard WD Hertz-Pannier L Mott SH Barnett AS Lebihan D Theodore WH. Functional anatomy of cognitive development: fMRI of verbal fluency in children and adults. Neurology (2000) 54:180–5. 10.1212/WNL.54.1.180 10636145

[18] Nair VA La C Nadkarni T Reiter P Chacon M Jensen M Abstract TP50: Functional Recovery in Stroke: Performance on Verbal Fluency Task. Honolulu: American Heart Association, Inc (2013).

[19] Wagner S Sebastian A Lieb K Tuscher O Tadic A. A coordinate-based ALE functional MRI meta-analysis of brain activation during verbal fluency tasks in healthy control subjects. BMC Neurosci (2014) 15:19. 10.1186/1471-2202-15-19 24456150 PMC3903437

[20] Benton A Hamsher K Sivan A Multilingual Aphasia Examination. Iowa City, IA: AJA (1976).

[21] Brady CB Spiro A III Mcglinchey-Berroth R Milberg W Gaziano JM. Stroke risk predicts verbal fluency decline in healthy older men: evidence from the normative aging study. J Gerontol B Psychol Sci Soc Sci (2001) 56:340–6. 10.1093/geronb/56.6.P340 11682587

[22] Philipose LE Alphs H Prabhakaran V Hillis AE. Testing conclusions from functional imaging of working memory with data from acute stroke. Behav Neurol (2007) 18:37–43. 10.1155/2007/396946 17297218 PMC5469977

[23] Prabhakaran V Raman SP Grunwald MR Mahadevia A Hussain N Lu H Neural substrates of word generation during stroke recovery: the influence of cortical hypoperfusion. Behav Neurol (2007) 18:45–52. 10.1155/2007/430402 17297219 PMC5469951

[24] Cox RW AFNI: software for analysis and visualization of functional magnetic resonance neuroimages. Comput Biomed Res (1996) 29:162–73. 10.1006/cbmr.1996.0014 8812068

[25] Cramer SC Nelles G Benson RR Kaplan JD Parker RA Kwong KK A functional MRI study of subjects recovered from hemiparetic stroke. Stroke (1997) 28:2518–27. 10.1161/01.STR.28.12.2518 9412643

[26] Saur D Ronneberger O Kummerer D Mader I Weiller C Kloppel S. Early functional magnetic resonance imaging activations predict language outcome after stroke. Brain (2010) 133:1252–64. 10.1093/brain/awq021 20299389

[27] Saur D Hartwigsen G. Neurobiology of language recovery after stroke: lessons from neuroimaging studies. Arch Phys Med Rehabil (2012) 93:S15–25. 10.1016/j.apmr.2011.03.036 22202187

[28] Pineiro R Pendlebury S Johansen-Berg H Matthews PM. Altered hemodynamic responses in patients after subcortical stroke measured by functional MRI. Stroke (2002) 33:103–9. 10.1161/hs0102.100482 11779897

[29] Lipp I Murphy K Caseras X Wise RG. Agreement and repeatability of vascular reactivity estimates based on a breath-hold task and a resting state scan. Neuroimage (2015) 113:387–96. 10.1016/j.neuroimage.2015.03.004 25795342 PMC4441043

[30] D’Esposito M Deouell LY Gazzaley A Alterations in the BOLD fMRI signal with ageing and disease: a challenge for neuroimaging. Nat Rev Neurosci (2003) 4:863–72. 10.1038/nrn1246 14595398

[31] Birn RM The role of physiological noise in resting-state functional connectivity. Neuroimage (2012) 62(2): 864–70. 10.1016/j.neuroimage.2012.01.016 22245341 PMC13374118

